# 11α-hydroxyprogesterone dampens lung metastasis *via* EMT modulation in PyMT-induced breast cancer murine model

**DOI:** 10.1186/s42826-025-00259-1

**Published:** 2025-10-14

**Authors:** Narim Kim, Jinhee Lee, Ah Young Song, Moeka Mukae, Beum-Soo An, Eui-Ju Hong

**Affiliations:** 1 College of Veterinary Medicine, Chungnam National University, Suite 401, Veterinary Medicine Bldg., 99, Daehak-ro, Yuseong-gu, Daejeon, 34134 Republic of Korea; 2 Department of Biomaterials Science, College of Natural Resources & Life Science, Pusan National University, Miryang, 50463 Republic of Korea

**Keywords:** 11α-OHP, Breast cancer, Lung, Metastasis, Migration

## Abstract

**Background:**

Despite the availability of various therapeutic strategies, the prognosis for patients with metastatic breast cancer remains poor. Epithelial-mesenchymal transition (EMT) is a critical mechanism driving metastasis in breast cancer, enabling tumor cells to lose epithelial characteristics and acquire enhanced motility and invasiveness.

**Results:**

This study investigates the role of 11alpha-hydroxyprogesterone (11α-OHP), a steroid hormone with an incompletely understood biosynthesis and metabolic pathway, in regulating lung metastasis in breast cancer. Using the MMTV-PyMT FVB mouse model, which spontaneously develops breast tumors we administered 11α-OHP for five weeks starting at 10 weeks of age. At 15 weeks, histological analysis revealed a significant reduction in lung metastasis in 11α-OHP-treated mice compared to controls, with notably smaller metastatic tumor areas in the lungs. Additionally, treated mice exhibited increased expression of epithelial cell adhesion proteins and decreased levels of focal adhesion kinase (FAK) in lung tissues. In vitro experiments using MDA-MB-231 cells corroborated these findings, showing that 11α-OHP significantly inhibited cell motility and invasiveness in scratch wound, transwell migration, and invasion assays. Notably, 11α-OHP did not significantly alter primary tumor growth in the MMTV-PyMT model.

**Conclusions:**

These findings suggest that 11α-OHP may suppress breast cancer metastasis by modulating EMT, highlighting its potential as a therapeutic target for preventing metastatic progression.

**Supplementary Information:**

The online version contains supplementary material available at 10.1186/s42826-025-00259-1.

## Background

Breast cancer is the most prevalent malignancy among women and a significant contributor to cancer-related mortality. Its development is influenced by a combination of genetic and environmental factors [1]. Despite significant advancements in early detection and therapeutic strategies, recurrence, particularly due to organ metastasis, remains a major challenge, adversely affecting survival rates [2]. Breast cancer cells have the capability to disseminate to various distant organs, including the lungs, liver, bone, and brain, with approximately 80% of breast cancer-related deaths attributed to metastasis at these sites [3]. Notably, around 60% of patients with metastatic breast cancer develop lung metastases [4]. To improve treatment outcomes, it is essential to investigate the mechanisms underlying metastatic progression.

One critical mechanism in breast cancer is epithelial-to-mesenchymal transition (EMT), during which cancer cells lose their epithelial properties and gain motility and invasiveness. A key aspect of the EMT process is the downregulation of E-cadherin, a vital mediator of cell-cell adhesion. E-cadherin-mediated adhesion restricts cellular mobility and supports the establishment of apical-basal polarity [5]. The loss of E-cadherin disrupts tight junction integrity and induces a mesenchymal phenotype, facilitating the detachment of cancer cells from the primary tumor and their migration through the circulatory and lymphatic systems to distant sites, thus promoting metastasis. Additionally, the loss of apical-basal polarity leads to abnormal interactions between growth factors secreted from the apical domain and their homotypic receptors on the basolateral surface [6]. EMT is now widely recognized as a dynamic and reversible process rather than a binary switch, with tumor cells often existing in intermediate states along an EMT spectrum. These partial EMT states are associated with phenotypic plasticity and have been implicated in metastatic dissemination, particularly in breast cancer where lung metastasis is a frequent event [7]. Such transitional phenotypes allow cancer cells to adapt to diverse microenvironments, facilitating detachment, intravasation, and eventual colonization of distant organs.

The prognosis for cancer patients is predominantly determined by the presence of metastatic tumors rather than by the size or growth of the primary tumor [8]. Following EMT, cancer cells that migrate to the lungs can establish micro-metastases, which may eventually progress to macro-metastases. This progression is clinically associated with symptoms such as pain, coughing, hemoptysis, and compromised pulmonary function, all of which significantly influence patient survival [2]. To elucidate the molecular mechanisms governing lung metastasis in breast cancer, the utilization of transgenic mouse models that recapitulate human breast cancer pathology and develop lung metastases is critical.

In this study, we specifically examine lung metastasis utilizing the MMTV-PyMT mouse model. This model is characterized by the mammary epithelium-specific expression of PyMT, driven by the MMTV promoter, leading to multifocal mammary adenocarcinomas and metastatic lesions in the lungs and lymph nodes [9]. Tumor progression in this model encompasses four stages—hyperplasia, adenoma, early carcinoma, and late carcinoma—closely paralleling the progression observed in human breast cancer [10]. Furthermore, this model exhibits a short latency, high penetrance, and a significant incidence of lung metastasis [11]. Remarkably, within a few weeks, the MMTV-PyMT model replicates the intricate stages and heterogeneity of human breast cancer, rendering it a suitable platform for investigating breast cancer metastasis.

11α-OHP, a steroid hormone, is produced from progesterone via the action of the enzyme 11α-hydroxylase. However, the biosynthesis and metabolic pathways of 11α-OHP remain inadequately characterized [12, 13]. In the present study, we employed transgenic MMTV-PyMT FVB mice and investigated the potential therapeutic value of 11α-OHP in addressing breast cancer lung metastasis.

## Methods

### Animal

Transgenic PyMT (FVB/N-Tg(MMTV-PyVT)634Mul/J) mice were obtained from The Jackson Laboratory (022974). Female MMTV-PyMT FVB mice, aged 10 weeks, were housed under controlled conditions at Chungnam National University, maintaining a 12-hour light/dark cycle and a regulated temperature. The mice had unrestricted access to a standard diet and water. Over a 5-week period, the mice were subcutaneously implanted with pelletized 11α-OHP (60 mg/mouse), which was procured from Tokyo Chemical Industry (TCI, H0498). Mice were monitored weekly to track changes in body weight and every three days to assess the development of primary breast tumors and lung metastases. All animal experiments were approved by the Chungnam Facility Animal Care Committee (202307 A-CNU-126).

### Cell culture

MDA-MB-231 human breast cancer cells were cultured at 37 °C in a 5% CO2 atmosphere using Dulbecco’s Modified Eagle Medium (Welgene, LM001-05), supplemented with penicillin (100 U/mL), streptomycin (100 µg/mL), ciprofloxacin (10 µg/mL), gentamicin (50 µg/mL), and 5% heat-inactivated fetal bovine serum (FBS). To deplete endogenous steroid hormones, charcoal-dextran-treated FBS (CD-FBS) was used in phenol red-free medium (Welgene, LM002–05). Steroid depletion was performed by incubating cells in phenol red-free medium containing 2% CD-FBS for 24 h prior to the experiment.

### Cell scratch, transwell migration and invasion assays assays

Cell migration was assessed using the scratch assay, where straight scratches were made in the cell monolayer using micropipette tips. The scratched areas were marked for consistent measurement. Migration and invasion assays were performed using transwell plates. Cells that migrated through the hanging inserts were stained with crystal violet and counted in four fields under a microscope. Migration and invasion were quantified using ImageJ software.

### Cell proliferation and cytotoxicity assay

Cell viability was assessed using the EZ-Cytox assay (DoGenBio, EZ-500). MDA-MB-231 cells were seeded in 96-well plates, and EZ-Cytox reagent was added according to the manufacturer’s protocol. After 2 h of incubation in a CO2 incubator, absorbance was measured at 450 nm using a plate reader.

### Protein extraction and Western blotting analysis

Total protein was extracted from tumors and MDA-MB-231 cells using T-PER buffer supplemented with the protease inhibitor phenylmethylsulfonyl fluoride (Sigma-Aldrich, P7626). Protein concentrations were determined using a protein assay solution (iNtRON Biotechnology). Samples were boiled for 5 min at 100 °C prior to sodium dodecyl sulfate-polyacrylamide gel electrophoresis (SDS-PAGE). Proteins were transferred to membranes for 75 min at 350 mA, followed by blocking in 3% bovine serum albumin (BSA) for 30 min. Membranes were incubated overnight at 4 °C with primary antibodies diluted in 3% BSA. After washing with TBST, membranes were incubated with secondary antibodies (goat anti-rabbit IgG or goat anti-mouse IgG) diluted in 5% skim milk at 4 °C overnight. Protein detection was performed using an enhanced chemiluminescence solution (Cynagen, Italy). Primary antibodies included mouse anti-β-Actin, mouse anti-α-Tubulin (Proteintech, 6603-1-Ig), the EMT IF Antibody Sampler Kit (CST, 49398), rabbit anti-PCNA (13110, CST), rabbit anti-phospho-AKT (4060, CST), rabbit anti-phospho-ERK (9789, CST), rabbit anti-cleaved caspase-3 (CST, 9661), and rabbit anti-PARP (9532, CST).

### Hematoxylin and Eosin (H&E) staining

For H&E staining, paraffin-embedded tissue blocks were cut into 4–5 μm sections and mounted on silane-coated slides. The slides were deparaffinized in xylene and rehydrated through a series of ethanol (100% to 70%) followed by tap water. Sections were incubated in hematoxylin for 5 min for nuclear staining, followed by a 10-minute wash and a 1-minute incubation in eosin for cytoplasmic staining. The slides were then dehydrated using ethanol (70% to 100%) and xylene before being mounted with a cover slip.

### Immunofluorescence

For immunofluorescence, paraffin blocks were sectioned into 4–5 μm slices and mounted on silane-coated slides. Slides were deparaffinized overnight in xylene and rehydrated through a series of ethanol (100% to 70%) and distilled water. Antigen retrieval was performed by incubating the slides in 0.1% sodium citrate buffer at 95 °C for 60 min. After cooling to room temperature, slides were washed with TBST for 10 min and blocked with 3% BSA. Primary antibodies, including E-cadherin, claudin-1 (CST, 49398), FAK (CST, 3285), and Ki67 (GeneTex, GTX16667), were applied overnight at 4 °C, followed by three washes with TBST.

### Statistical analysis

Data are presented as mean ± standard deviation. The differences between means were obtained through Student’s t-test and one-way ANOVA. All statistical analyses were performed using GraphPad Software (GraphPad Inc., San Diego, CA, USA).

## Results

### 11α-OHP does not decrease tumor development and proliferation in MMTV-PyMT mice

**Fig. 1:**
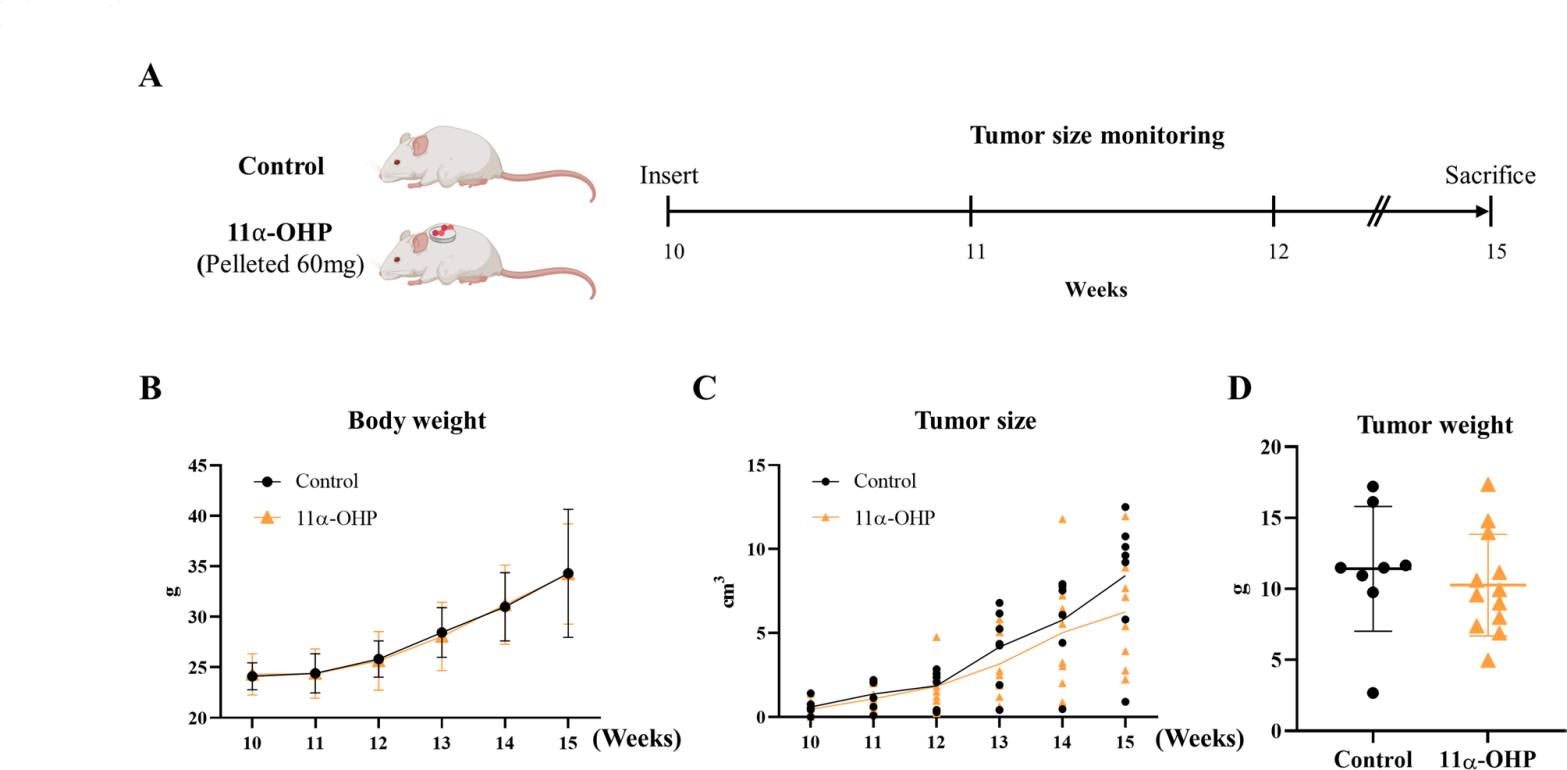
11α-OHP does not inhibit tumor development or proliferation in MMTV-PyMT mice. (**A**) Experimental timeline. MMTV-PyMT mice were treated with 11α-OHP for 5 weeks. This image was created with BioRender.com*(#IM27O9Z2CO*). (**B**) Monitoring weight changes in control mice (*n* = 8) and 11α-OHP-treated mice (*n* = 12) over the course of the experiment. (**C**) Tumor size progression in control mice (*n* = 8) and 11α-OHP-treated mice (*n* = 12) during the experimental period. (**D**) Tumor weight of control mice (*n* = 8) and 11α-OHP-treated mice (*n* = 12) at 15 weeks. Statistical analysis was performed using Student’s t-test. Values are presented as means ± SD

Building on a previous study in which most mice developed palpable nodules around 10 weeks of age [14], we subcutaneously implanted pelletized 11α-OHP into 10-week-old MMTV-PyMT mice to investigate its role in breast cancer. The mice were sacrificed 5 weeks later, and the results were compared to those of control mice at 15 weeks (Fig. 1A). Throughout the experimental period, body weight was monitored for both mice, showing no significant differences (Fig. 1B). Tumor sizes were also recorded, revealing no significant variations between two groups (Fig. 1C). At 15 weeks of age, the average tumor weights were comparable between the control and 11α-OHP treated mice (*p =* 0.5287; Fig. 1D). Overall, these findings indicate that 11α-OHP did not significantly affect tumor growth in MMTV-PyMT mice.

**Fig. 2:**
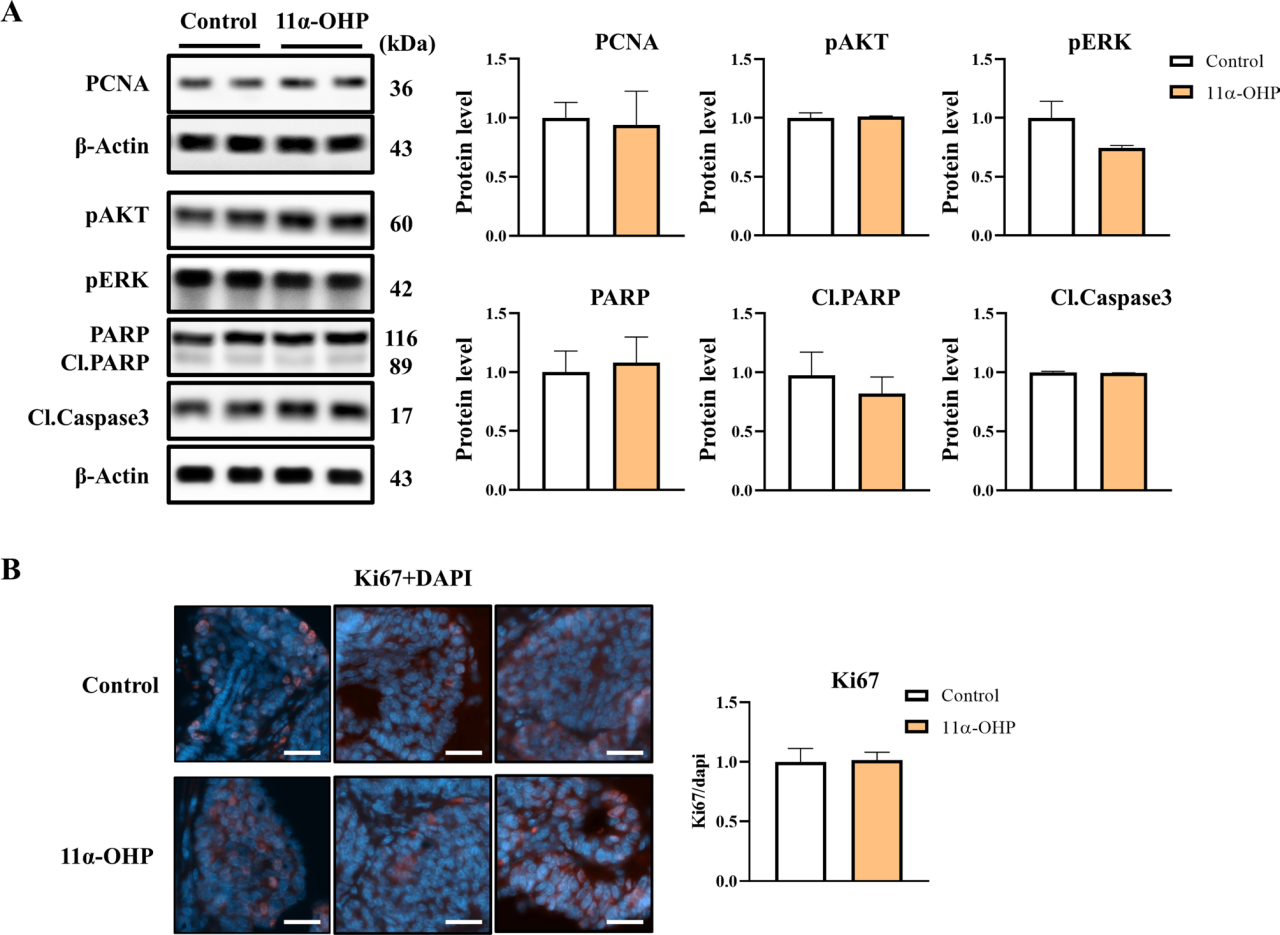
11α-OHP does not regulate the growth and proliferation of breast cancer in MMTV-PyMT mice. (**A**) Western blot analysis of proliferation-related proteins (PCNA, pAKT, and pERK) and apoptosis markers (cleaved PARP and cleaved caspase 3) in mammary tumors from control and 11α-OHP-treated mice at 15 weeks. β-Actin was used as an internal control for protein quantification. (**B**) Immunofluorescence staining for Ki67 in mammary tumors of control and 11α-OHP-treated mice at 15 weeks. The scale bar represents 25 μm. DAPI was used for nuclear staining. Statistical analysis was performed using Student’s t-test. Values are presented as means ± SD

The expression levels of proliferation markers, including proliferating cell nuclear antigen (PCNA), AKT, phospho-AKT, and phospho-ERK, showed no significant differences between the control and 11α-OHP treated mice (Fig. 2A). Additionally, the expression levels of apoptotic markers (PARP, cleaved PARP, and cleaved caspase-3) were also similar between two groups (Fig. 2A). Ki67 immunofluorescence analysis further confirmed no significant differences in tumor cell proliferation between the two groups (Fig. 2B). Thus, these results suggest that 11α-OHP does not play a significant role in tumor cell development, as indicated in Fig. 1C-D.

### 11α-OHP regulates emt markers and inhibits lung metastasis in MMTV-PyMT mice

**Fig. 3:**
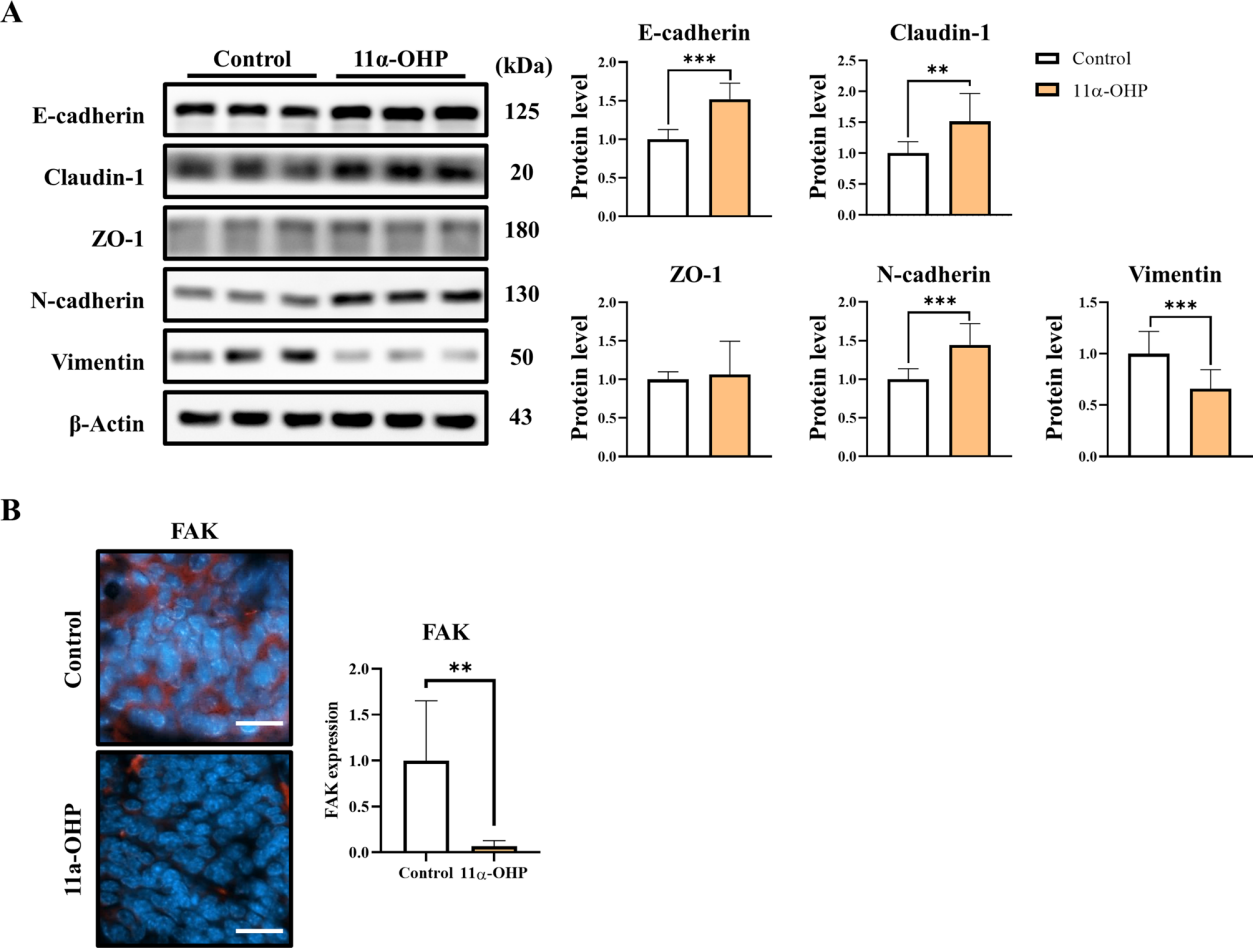
11α-OHP inhibits the loss of epithelial cell adhesion proteins in MMTV-PyMT mice. (**A**) Western blot analysis of epithelial-mesenchymal transition (EMT)-related markers in mammary tumors of control and 11α-OHP-treated mice at 15 weeks. Quantification was performed using β-Actin as an internal control. (**B**) Immunofluorescence staining for FAK in mammary tumors of control and 11α-OHP-treated mice at 15 weeks. Immunofluorescence quantification was performed using ImageJ. The scale bar represents 50 μm. DAPI was used for nuclear staining. Statistical analysis was performed using Student’s t-test. Values are presented as means ± SD. **p < 0.01, ***p < 0.001 versus control

Western blot analysis revealed that E-cadherin and claudin-1 protein levels were increased in the 11α-OHP treated mice (*p <* 0.001, 1.52-fold and *p <* 0.01, 1.51-fold compared with Control), while N-cadherin levels were elevated, and vimentin levels were significantly reduced compared to the control (*p <* 0.001, 1.44-fold and *p <* 0.001, 34%; Fig. 3A). To determine whether these effects also occur in normal tissue, we conducted an additional experiment using normal FVB mice. Mammary tissue was analyzed following short-term (3-day) exposure to 11α-OHP (2 mg/kg/day). Under these conditions, we observed similar expression changes in claudin-1, N-cadherin, and vimentin (Fig. S1). To further investigate these effects, we examined EMT-related protein expression in MDA-MB-231 cells treated with serum from Control and 11α-OHP-treated mice. The results showed an increase in E-cadherin protein levels (*p =* 0.07, 1.482-fold; Fig. S2).

Immunofluorescence studies indicated that FAK expression in tumors from the 11α-OHP treated mice decreased by 93.3% (*p <* 0.01) compared to the control mice (Fig. 3B). These results suggest that 11α-OHP may inhibit the loss of epithelial markers and reduce vimentin expression, thereby limiting tumor cell invasion and migration to other organs. Consistent with prior studies [15], which reported decreased lung metastasis in FAK-deficient MMTV-PyMT mice, our findings indicate that the 11α-OHP treated mice exhibited lower FAK expression than the control mice.

**Fig. 4:**
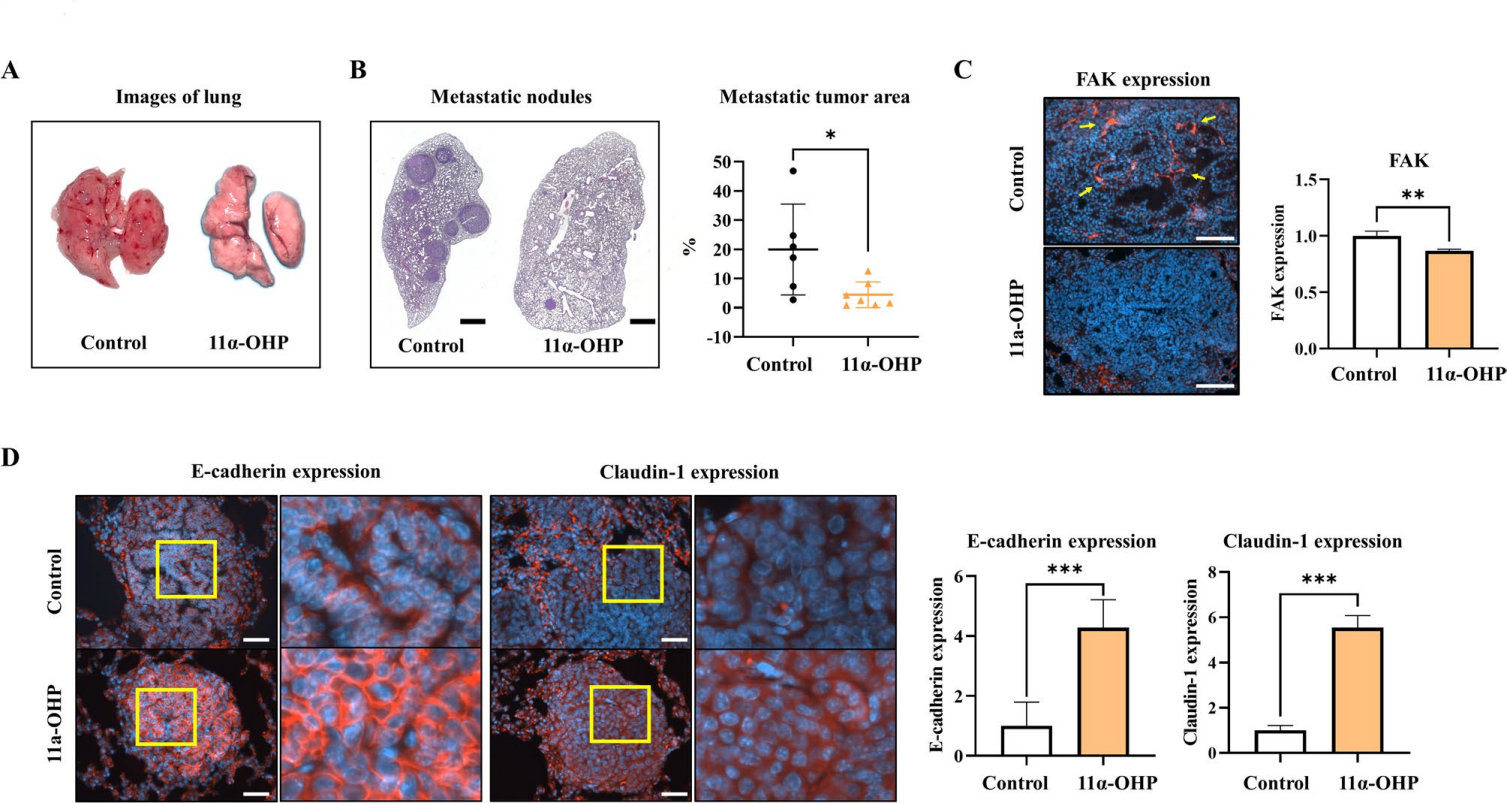
11α-OHP inhibits lung metastasis of breast cancer in MMTV-PyMT mice. (**A**) Representative lung tissues from control and 11α-OHP-treated mice at 15 weeks. (**B**) H&E staining of lung tissues from control and 11α-OHP-treated mice at 15 weeks. Metastatic tumors are stained dark purple. The scale bar represents 1000 μm. The graph quantifies the area of metastatic tumors per lung lobe. (**C**) Immunofluorescence staining for FAK in metastatic tumors in the lungs of control and 11α-OHP-treated mice at 15 weeks. Quantification was performed using ImageJ with DAPI as an internal control. The scale bar represents 100 μm. (**D**) Immunofluorescence staining for EMT markers in metastatic tumors in the lungs of control and 11α-OHP-treated mice at 15 weeks. The scale bar represents 50 μm. Graphs show quantification of positive (red) areas per DAPI (blue). Statistical analysis was performed using Student’s t-test. Values are presented as means ± SD. *p < 0.05, **p < 0.01, ***p < 0.001 versus control

Interestingly, the incidence of lung metastasis was significantly reduced in the 11α-OHP-treated mice (*p <* 0.05, 15.5% compared with Control mice) (Fig. 4A). Histological examination of lung tissues stained with hematoxylin and eosin revealed a higher level of metastatic tumors in the control mice compared to the 11α-OHP treated mice (Fig. 4B). Additionally, immunofluorescence analysis showed that FAK expression in lung tissues decreased in the 11α-OHP treated mice compared to the control mice (*p <* 0.01, 13%; Fig. 4C). Furthermore, the expression levels of epithelial cell adhesion proteins, including E-cadherin and claudin-1, were greater in the 11α-OHP treated mice than in the control mice (*p <* 0.001, 4.27-fold and 5.54-fold; Fig. 4D). These findings indicate that 11α-OHP suppresses the loss of epithelial adhesion proteins in metastatic tumors.

### 11α-OHP downregulates migration and invasion of MDA-MB-231 cells

**Fig. 5:**
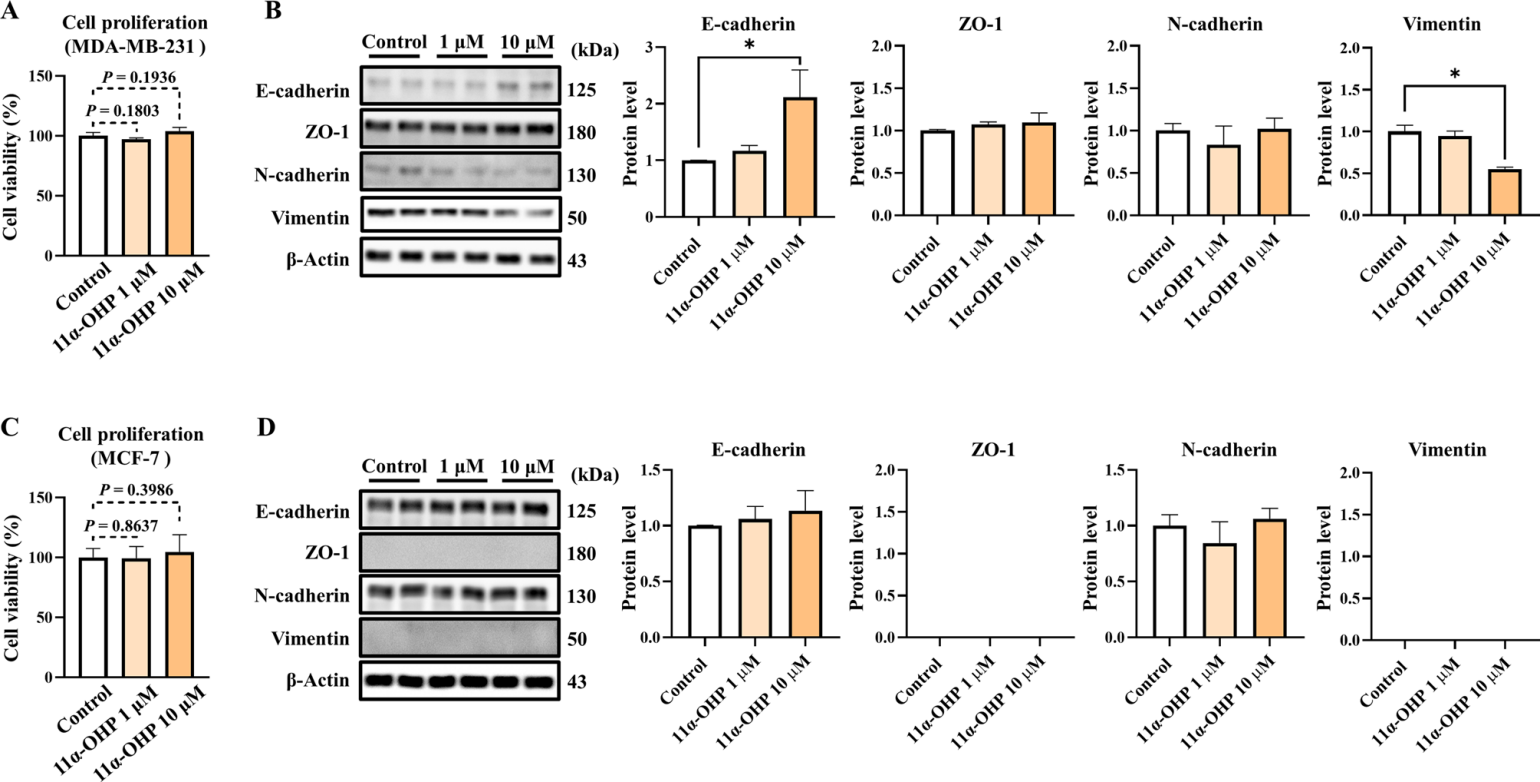
11α-OHP does not regulate cell proliferation and apoptosis in MDA-MB-231 cells. (**A**) Cell viability of MDA-MB-231 cells treated with 11α-OHP, as measured by the EZ-Cytox assay. (**B**) Western blot analysis of EMT markers (E-cadherin, ZO-1, N-cadherin, and vimentin) in MDA-MB-231 cells, with β-Actin used as an internal control. (**C**) Cell viability of MCF-7 cells treated with 11α-OHP, as measured by the EZ-Cytox assay. (**D**) Western blot analysis of EMT markers (E-cadherin, ZO-1, N-cadherin, and vimentin) in MCF-7 cells, with β-Actin used as an internal control. Statistical analysis was performed using Student’s t-test and one-way ANOVA. Values are presented as means ± SD. *p < 0.05 versus control

MDA-MB-231 cells were preincubated for 24 h in charcoal dextran-treated medium to deplete steroid hormones, followed by a 24-hour incubation with either vehicle (DMSO) or 11α-OHP (Fig. 5A-C). To assess the cytotoxic and proliferative effects of 11α-OHP, we conducted an EZ-Cytox assay (Fig. 5A). The expression levels of EMT-related markers were significantly different in the 11α-OHP treated cells compared to the control cells, especially at 10 µM. In the 10 µM treated cells, E-cadherin increased by 2.11-fold compared to control, and vimentin decreased by 44.9% compared to control (Fig. 5B). Furthermore, we performed the same experiment in MCF-7 cells, which represent the luminal-A subtype, but observed no significant differences in either E-cadherin or N-cadherin expression (Fig. 5C–D). Unfortunately, ZO-1 and vimentin expression levels were barely detectable in these cells.

**Fig. 6:**
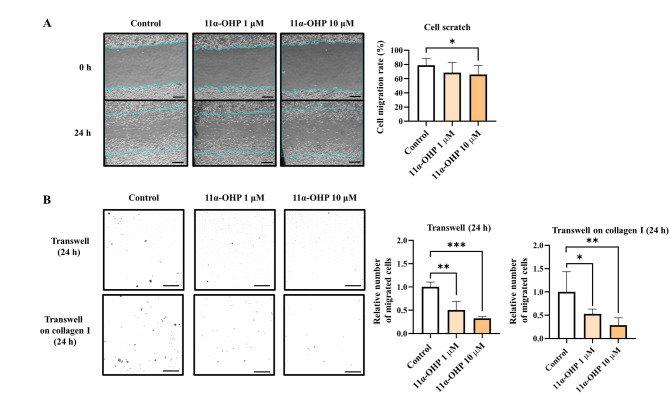
11α-OHP decreases migration and invasion in MDA-MB-231 cells. (**A**) Cell scratch assay to assess migration of MDA-MB-231 cells treated with either vehicle (DMSO) or 11α-OHP (dissolved in DMSO). The scale bar represents 40 μm. (**B**) Transwell migration and invasion assays in MDA-MB-231 cells. Cells were incubated for 24 h following treatment. Cells were counted in four separate fields using a microscope (scale bar: 20 μm). Statistical analysis was performed using Student’s t-test. Values are presented as means ± SD. *p < 0.05, **p < 0.01, ***p < 0.001 versus control

To evaluate the migratory activity of breast cancer cells, we performed a cell scratch assay and transwell migration assay using MDA-MB-231 cells. The degree of migration decreased in 11α-OHP treated cells (*p <* 0.05, 13.2%) (Fig. 6A). In the transwell migration assay, 11α-OHP-treated cells exhibited reduced migration compared to the control cells, with a 49.6% reduction at 1 µM (*p <* 0.01) and a 67.3% reduction at 10 µM (*p <* 0.001) (Fig. 6B). We also investigated the invasion ability of 11α-OHP-treated MDA-MB-231 cells in a transwell assay coated with collagen I. The results showed a decreased invasion rate in 11α-OHP-treated cells compared to controls, with reductions of 47.2% at 1 µM (*p <* 0.05) and 71.3% at 10 µM (*p <* 0.01) (Fig. 6B). These results suggest that 11α-OHP dampens cell mobility by increasing E-cadherin in MDA-MB-231 cells.

## Discussion

Lung metastasis remains a leading cause of mortality in breast cancer patients. Unlike primary tumors, which are often amenable to surgical removal, metastatic disease presents a significantly greater therapeutic challenge due to the complexity of the metastatic cascade, including cancer cell migration, invasion, and colonization of distant organs. Although the MMTV-PyMT transgenic mouse model does not fully recapitulate the phenotypic heterogeneity of metastatic breast cancer, it is widely regarded as a robust and reliable in vivo system for studying lung metastasis. In this study, we used 11α-OHP, a naturally occurring steroid hormone and a metabolite of progesterone. It functions as a potent inhibitor of 11β-hydroxysteroid dehydrogenase (11β-HSD), an enzyme involved in cortisol metabolism [16]. While it is not classified as an androgen, estrogen, or progestogen, 11α-OHP could influence the activity of other steroid hormones. Although the specific receptor or molecular target of 11α-OHP remains unidentified, our findings demonstrate that 11α-OHP can modulate lung metastasis in the PyMT-induced murine model of breast cancer.

After confirming tumor development around 10 weeks of age, we subcutaneously implanted pelletized 11α-OHP (60 mg) for 5 weeks. By the time the mice were sacrificed at 15 weeks old, the pellets were nearly fully absorbed and had disappeared from the body. Although there is no established data on 11α-OHP exposure in mouse models, we estimated the dosage based on earlier studies [17, 18], selecting a 5-week treatment duration to approximate progesterone levels (2 mg/mouse per day) used in those studies. Throughout the experimental period, the condition of both groups of mice was monitored, with no significant signs of toxicity or safety concerns observed. We assessed the expression levels of key proliferation markers-including PCNA, phosphorylated AKT (pAKT), and phosphorylated ERK (pERK)-as well as apoptosis markers such as cleaved PARP and cleaved caspase-3, in the tumors of MMTV-PyMT mice. Given that PCNA is essential for efficient DNA replication [19], and that pAKT and pERK are involved in cell proliferation during tumor progression [20], our results showed no significant differences in the expression of these proliferation markers between control and 11α-OHP-treated groups. Similarly, the expression of Ki67, a well-established nuclear marker of actively dividing cells [21], did not significantly differ between the two groups, consistent with the Western blot analysis.

Interestingly, we observed increased expression of epithelial markers, such as E-cadherin and Claudin-1, in the tumors of 11α-OHP-treated mice. Previous studies have indicated that E-cadherin and Claudin-1 act as suppressors of breast tumorigenesis [22–25]. In contrast, while N-cadherin levels were also elevated, we observed a marked decrease in vimentin expression, suggesting that 11α-OHP may affect cells in an intermediate EMT state, which often exhibit both epithelial and mesenchymal traits [26, 27]. Complete EMT typically involves a switch from cytokeratin to vimentin in intermediate filaments; however, cells in this intermediate state do not necessarily lose all epithelial characteristics, and the transition from cytokeratin to vimentin can facilitate cell migration. Our findings suggest that 11α-OHP treatment reduces vimentin expression in these intermediate-state cells, which retain both epithelial and mesenchymal features.

A notable reduction in lung metastasis was observed in the 11α-OHP treatment mice. Histological examination using H&E staining demonstrated a significant difference in the area occupied by metastatic tumors in the lungs between the 11α-OHP treated mice and controls. Furthermore, the focal adhesion kinase (FAK) protein levels, which have been associated with diminished lung metastasis in MMTV-PyMT mice when downregulated [15], were significantly reduced in the 11α-OHP treated mice. Immunofluorescence analysis further revealed increased expression of the epithelial adhesion proteins E-cadherin and claudin-1 in the lungs of the 11α-OHP-treated mice compared to controls. These findings support previous research indicating that preventing the loss of E-cadherin can inhibit the extravasation and subsequent metastasis of cancer cells to lung tissue [28].

E-cadherin has been widely recognized as a tumor suppressor protein and is frequently downregulated during EMT, a key process that facilitates cancer cell metastasis [23]. The loss of E-cadherin-mediated cell–cell adhesion is a critical event in tumor invasion and metastatic progression [29]. Our results indicate that treatment with 11α-OHP may prevent the downregulation of E-cadherin, thereby delaying cancer cell migration and invasion. These findings suggest that 11α-OHP may hinder the mesenchymal transition of cancer cells, ultimately reducing their motility and invasiveness. In vivo, it is possible that 11α-OHP exerts its anti-metastatic effects either by directly inhibiting EMT-related pathways or by indirectly modulating tumor cell behavior through enhancing the activity or availability of cortisol from adrenal gland [16]. To distinguish between these mechanisms, we performed in vitro cell-based assays under controlled conditions to eliminate systemic hormonal influences. We utilized MDA-MB-231 cells, a highly aggressive, invasive, and poorly differentiated triple-negative breast cancer cell line lacking ER, PR, and HER2 amplification. These cells are characterized by EMT-associated markers and exhibit an invasive phenotype, often displaying star-shaped projections that connect multiple cell clusters [30]. Consistent with our in vivo findings, no significant differences were observed in proliferation or apoptosis marker expression between control and 11α-OHP-treated cells. However, we detected marked changes in EMT marker expression: 11α-OHP treatment directly increased E-cadherin levels while reducing vimentin expression, closely mirroring the effects seen in vivo. Given that vimentin knockdown has been shown to significantly impair cell motility in MDA-MB-231 cells [31], these results suggest that 11α-OHP may suppress the migratory potential of these cells by modulating EMT-related pathways.

To further support our molecular findings, we conducted cell scratch and transwell migration assays, both of which demonstrated reduced migratory capacity in 11α-OHP-treated cells compared to controls. Additionally, to assess the effect of 11α-OHP on the invasive potential of MDA-MB-231 cells, we performed a transwell invasion assay using a collagen I matrix. Collagen I, a rope-like triple-helical structural protein found abundantly in the extracellular matrix (ECM), was selected to replicate the in vitro ECM environment [32]. MDA-MB-231 cells are known to invade collagen I through the activation of collagenase-1 [33]. In our invasion assays, the number of invading cells was significantly reduced in the 11α-OHP-treated group, indicating that 11α-OHP impairs the invasive capacity. Given that its loss facilitates the infiltration and dissemination of primary tumors through collagen I matrices [34], our findings suggest that 11α-OHP may reduce cell invasiveness by upregulating E-cadherin expression. This evidence may underlie the decreased invasiveness observed in our functional assays.

Although our in vivo (MMTV-PyMT) and in vitro (MDA-MB-231) models differ in molecular subtype, the consistent suppression of EMT-related markers by 11α-OHP across both systems strengthens the generalizability of our findings. The mouse model enabled the evaluation of spontaneous lung metastasis in a hormone receptor-positive context, while MDA-MB-231 cells served as a platform to assess direct modulation of EMT in a highly invasive, triple-negative background. Notably, in MCF-7 cells, which are exhibit less mesenchymal characteristics, 11α-OHP treatment did not significantly affect migration, likely due to the low expression of EMT proteins. These results suggest that the anti-metastatic effects of 11α-OHP may be more pronounced in aggressive, mesenchymal-like breast cancer cells. Collectively, our findings indicate that 11α-OHP may broadly inhibit metastatic traits, particularly in tumors with strong mesenchymal features, although further validation across diverse molecular subtypes is needed.

## Conclusions

While the precise mechanisms by which 11α-OHP influences lung metastasis remain to be fully elucidated, our findings suggest that 11α-OHP may attenuate the loss of E-cadherin during the EMT by promoting its expression. Collectively, these results indicate that enhancing 11α-OHP levels in breast cancer patients could represent a promising strategy to reduce pulmonary metastasis and improve patient survival, highlighting its potential as a therapeutic target for the prevention and treatment of metastatic breast cancer.

## Supplementary Information

Below is the link to the electronic supplementary material.

Supplementary Material 1

Supplementary Material 2

## Data Availability

The datasets used and/or analyzed during the current study are available from the corresponding author on reasonable request.

## Abbreviations

AKT: Protein kinase B
ECM: Extracellular matrix
EMT: Epithelial-mesenchymal transition
ERK: Extracellular regulated kinases
FAK: Focal adhesion kinase
PARP: Poly (ADP-ribose) polymerase
PCNA: Proliferating cell nuclear antigen
11α-OHP: 11α-Hydroxyprogesterone
