# Supplementary material for: 11α-hydroxyprogesterone dampens lung metastasis via EMT modulation in PyMT-induced breast cancer murine model
Source: Lab Anim Res. 2025 Oct 14;41:26. doi: 10.1186/s42826-025-00259-1 (PMC12519870; doi:10.1186/s42826-025-00259-1)
Supplement: Supplementary file 1 — Supplementary Material 1 [file 42826_2025_259_MOESM1_ESM.docx]

**Supplementary Materials**

11α-hydroxyprogesterone dampens lung metastasis *via* EMT modulation in PyMT-induced breast cancer murine model

Narim Kim^1^, Jinhee Lee^1^, Ah Young Song^1^, Moeka Mukae^1^, Beum-Soo An^2^, and Eui-Ju Hong^1*^

^1^College of Veterinary Medicine, Chungnam National University, Daejeon 34134, Republic of Korea; ^2^Department of Biomaterials Science, College of Natural Resources & Life Science, Pusan National University, Miryang 50463, Republic of Korea.

Corresponding authors: Eui-Ju Hong, DVM, PhD

College of Veterinary Medicine, Chungnam National University, Suite 401, Veterinary Medicine Bldg., 99, Daehak-ro, Yuseong-gu, Daejeon 34134, Korea.

Phone: +82-42-821-6781; Fax: +82-42-821-8903; Email:[ejhong@cnu.ac.kr](mailto:ejhong@cnu.ac.kr)

**
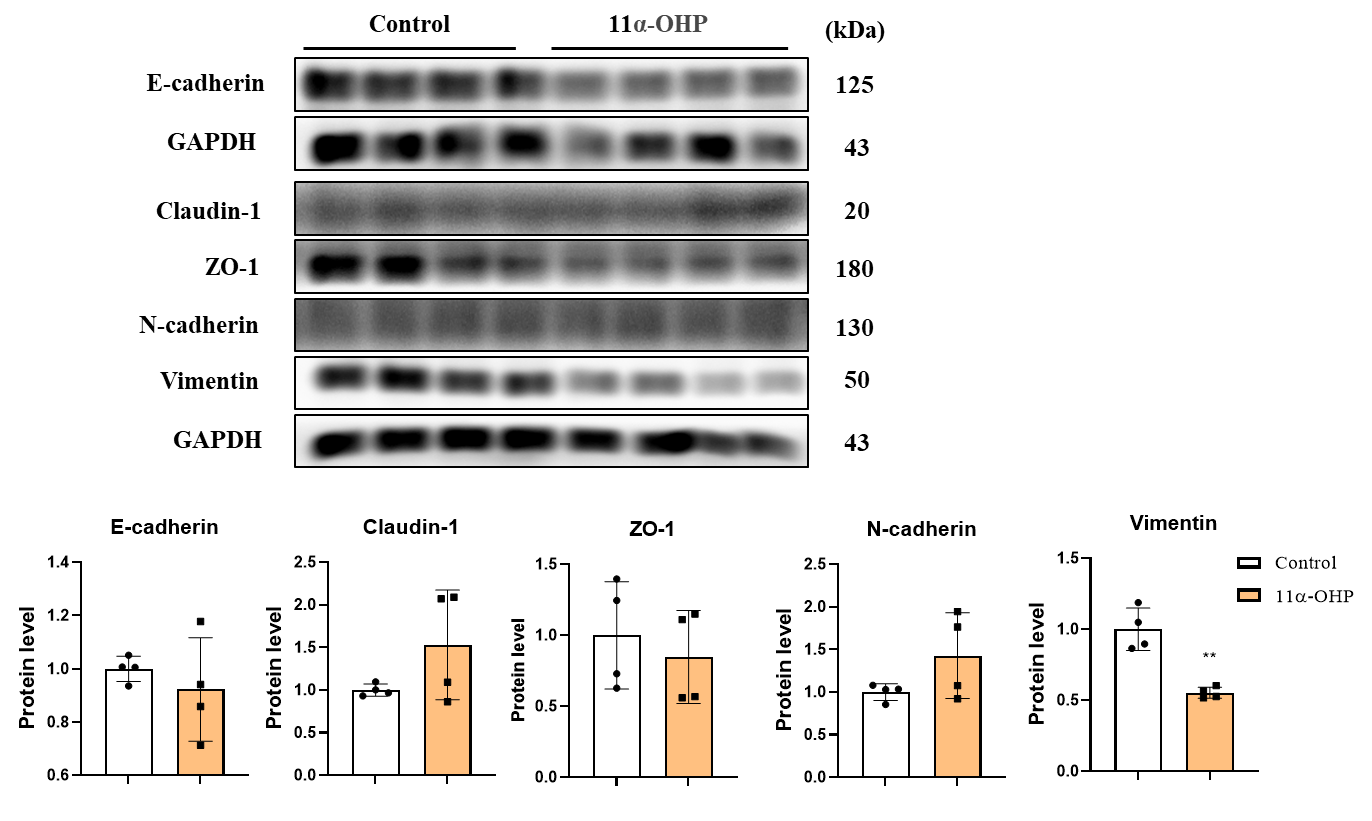
**

Supplementary figure 1. 11α-OHP fluctuate the epithelial or mesenchymal cell adhesion proteins in normal FVB mice. Western blot analysis of epithelial-mesenchymal transition (EMT)-related markers in normal breast tissue of control and 11α-OHP (2 mg/kg/day)-treated mice for 3 days. Quantification was performed using GAPDH as an internal control. Statistical analysis was performed using Student’s t-test. Values are presented as means ± SD. **p < 0.01 versus control.

**
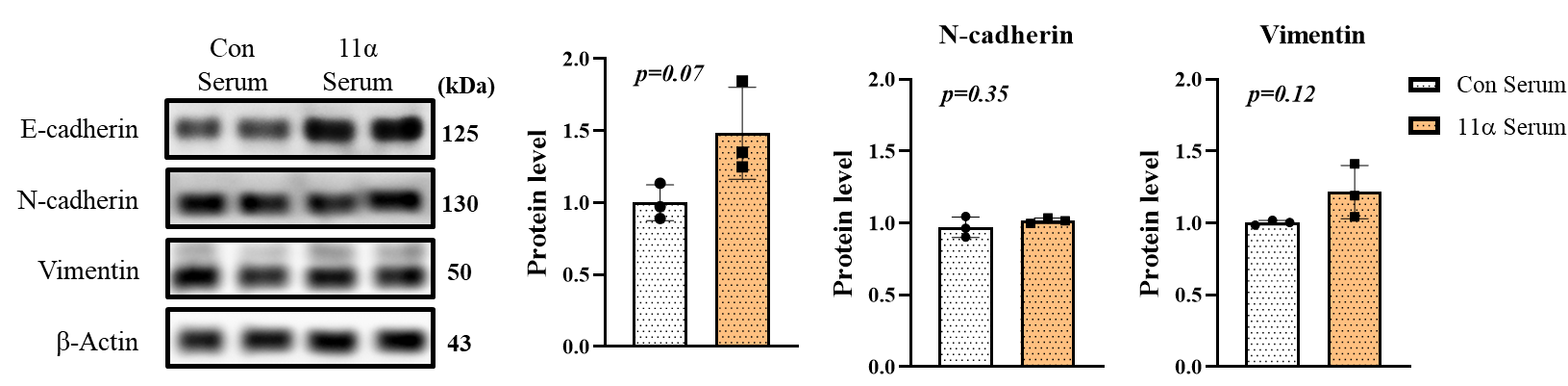
**

Supplementary figure 2. Effects of MMTV-PyMT mouse serum (Control and 11α-OHP-treated) on EMT-related protein expression in MDA-MB-231 cells after 24 hours. Western blot analysis was performed to examine the expression levels of EMT markers, including E-cadherin, N-cadherin, and vimentin, with β-actin used as an internal control. Statistical analysis was conducted using Student’s t-test, and the results are presented as means ± SD.
