# Supplementary material for: 11α-hydroxyprogesterone dampens lung metastasis via EMT modulation in PyMT-induced breast cancer murine model
Source: Lab Anim Res. 2025 Oct 14;41:26. doi: 10.1186/s42826-025-00259-1 (PMC12519870; doi:10.1186/s42826-025-00259-1)
Supplement: Supplementary file 2 — Supplementary Material 2 [file 42826_2025_259_MOESM2_ESM.pptx]

## Slide 1
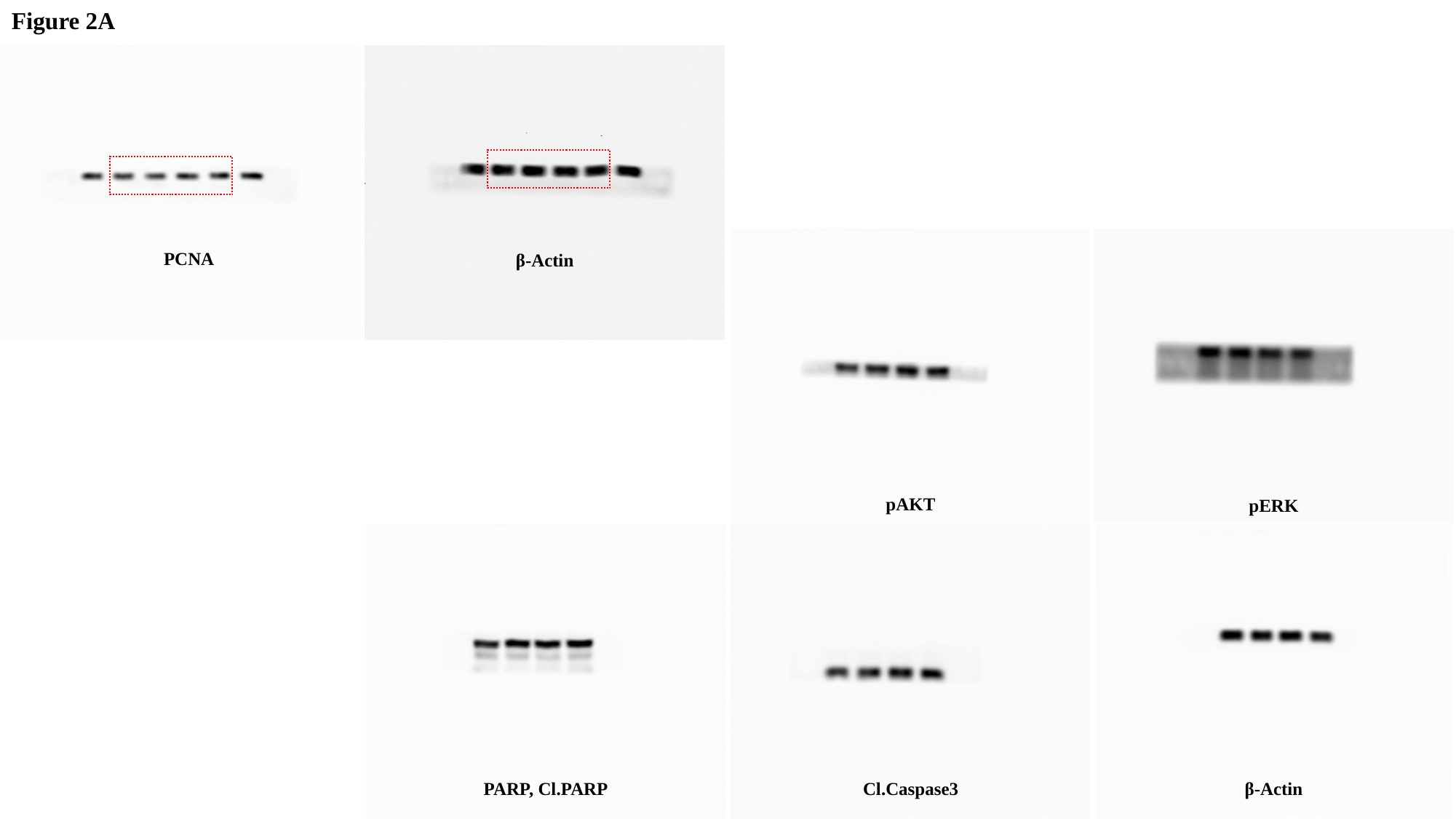

Figure 2A
PCNA
β-Actin
pAKT
pERK
Cl.Caspase3
β-Actin
PARP, Cl.PARP

## Slide 2
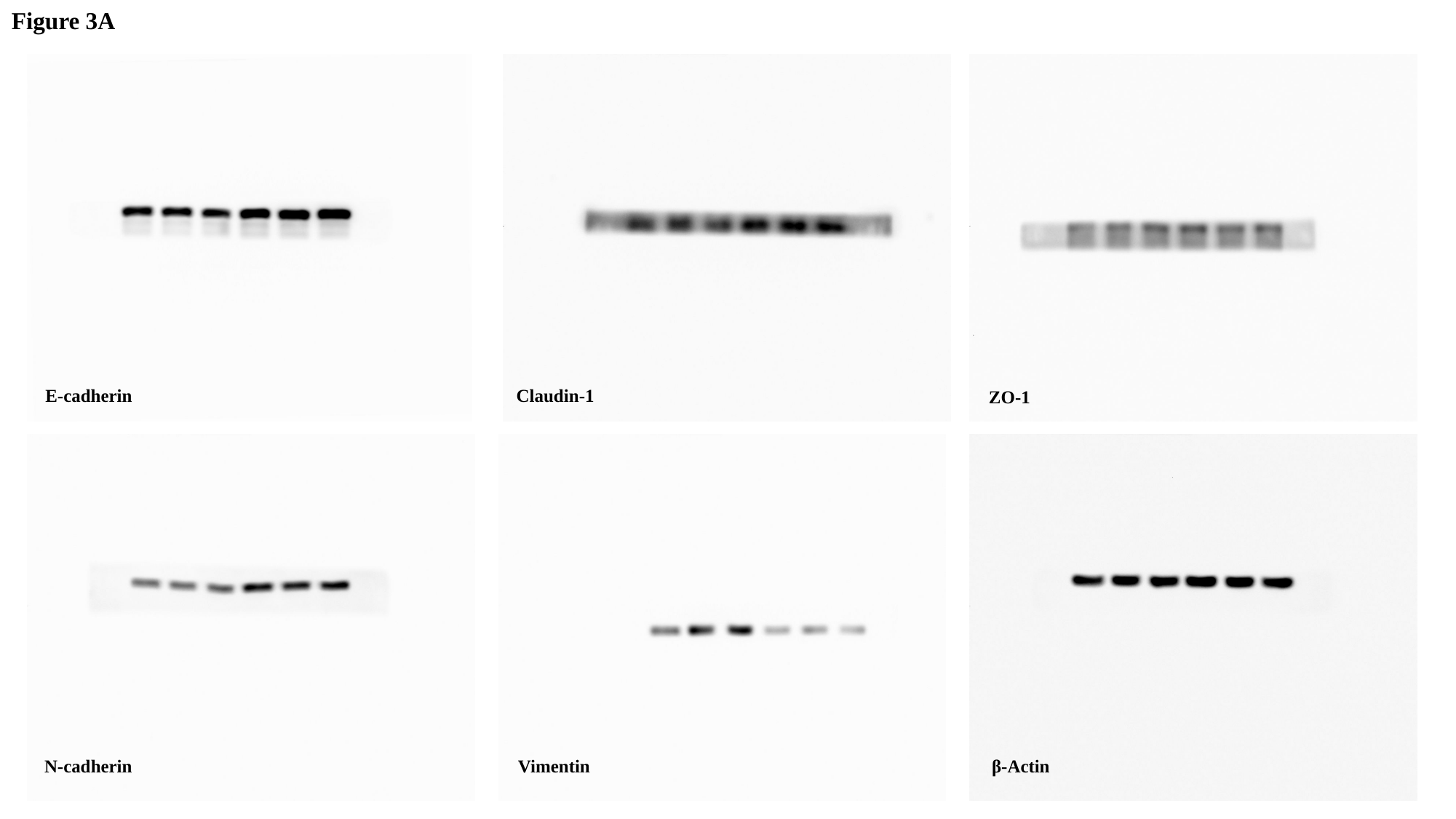

Figure 3A
E-cadherin
Claudin-1
ZO-1
N-cadherin
Vimentin
β-Actin

## Slide 3
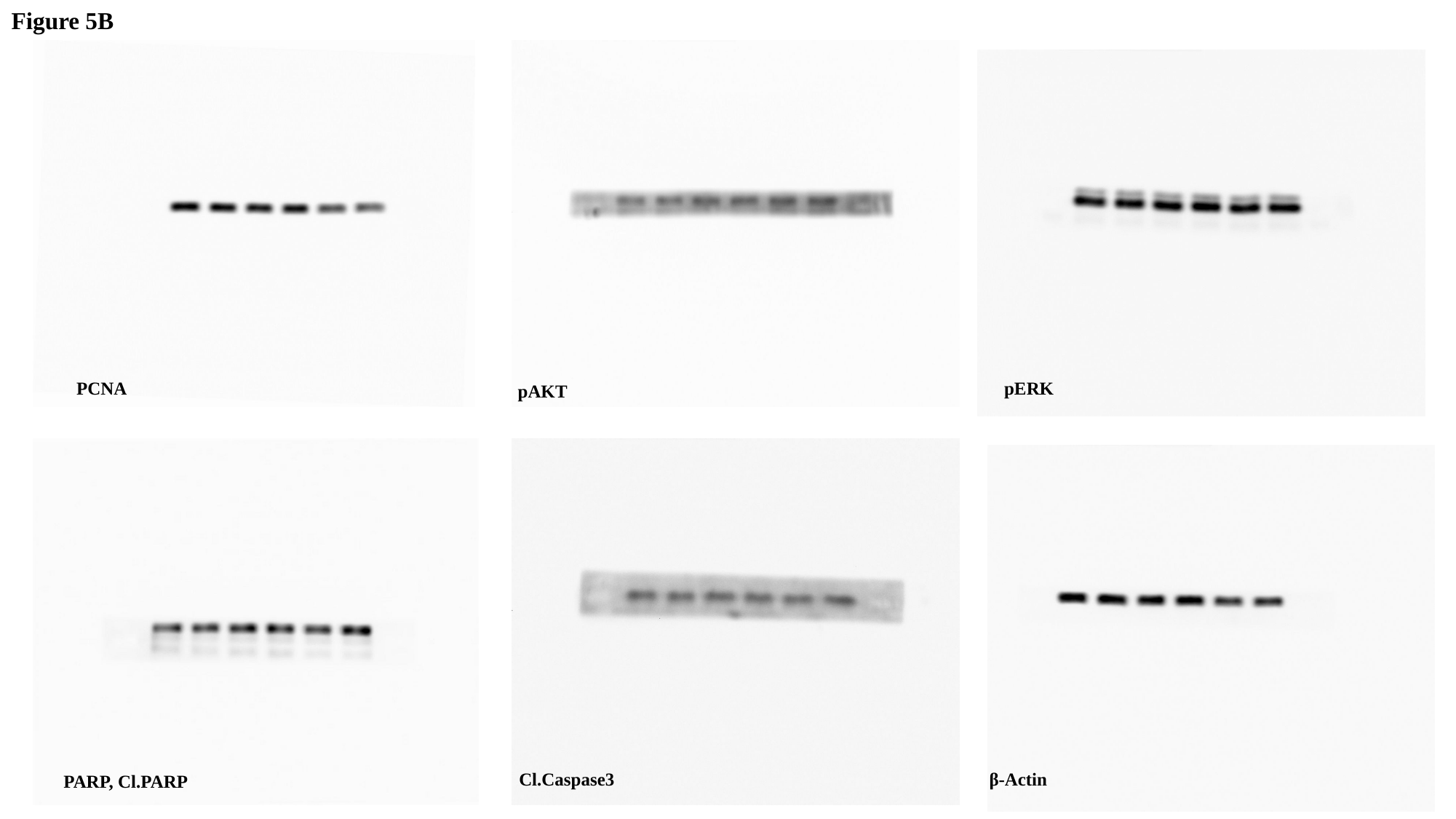

Figure 5B
PCNA
pERK
pAKT
Cl.Caspase3
β-Actin
PARP, Cl.PARP

## Slide 4
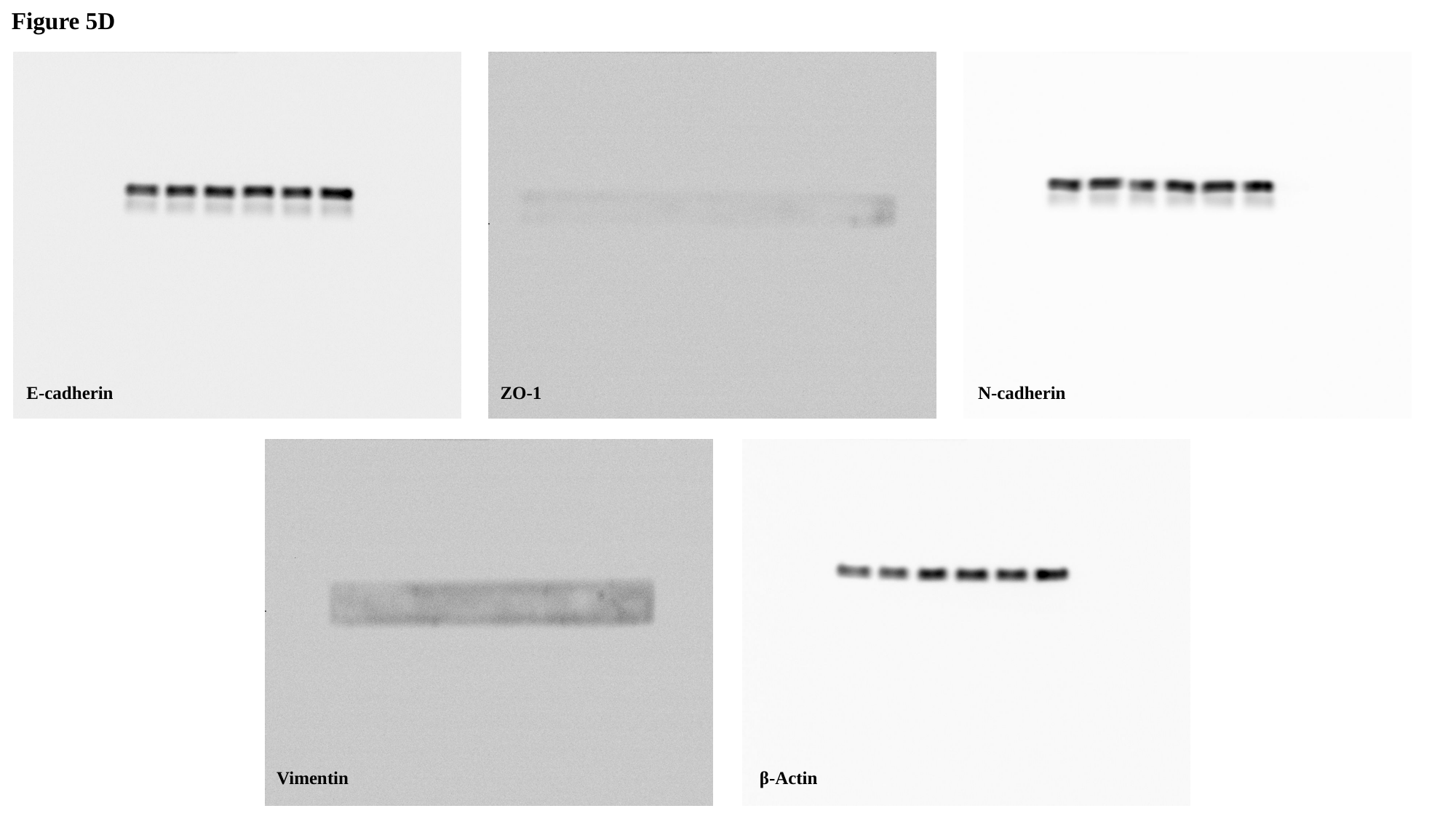

Figure 5D
ZO-1
N-cadherin
E-cadherin
β-Actin
Vimentin

## Slide 5
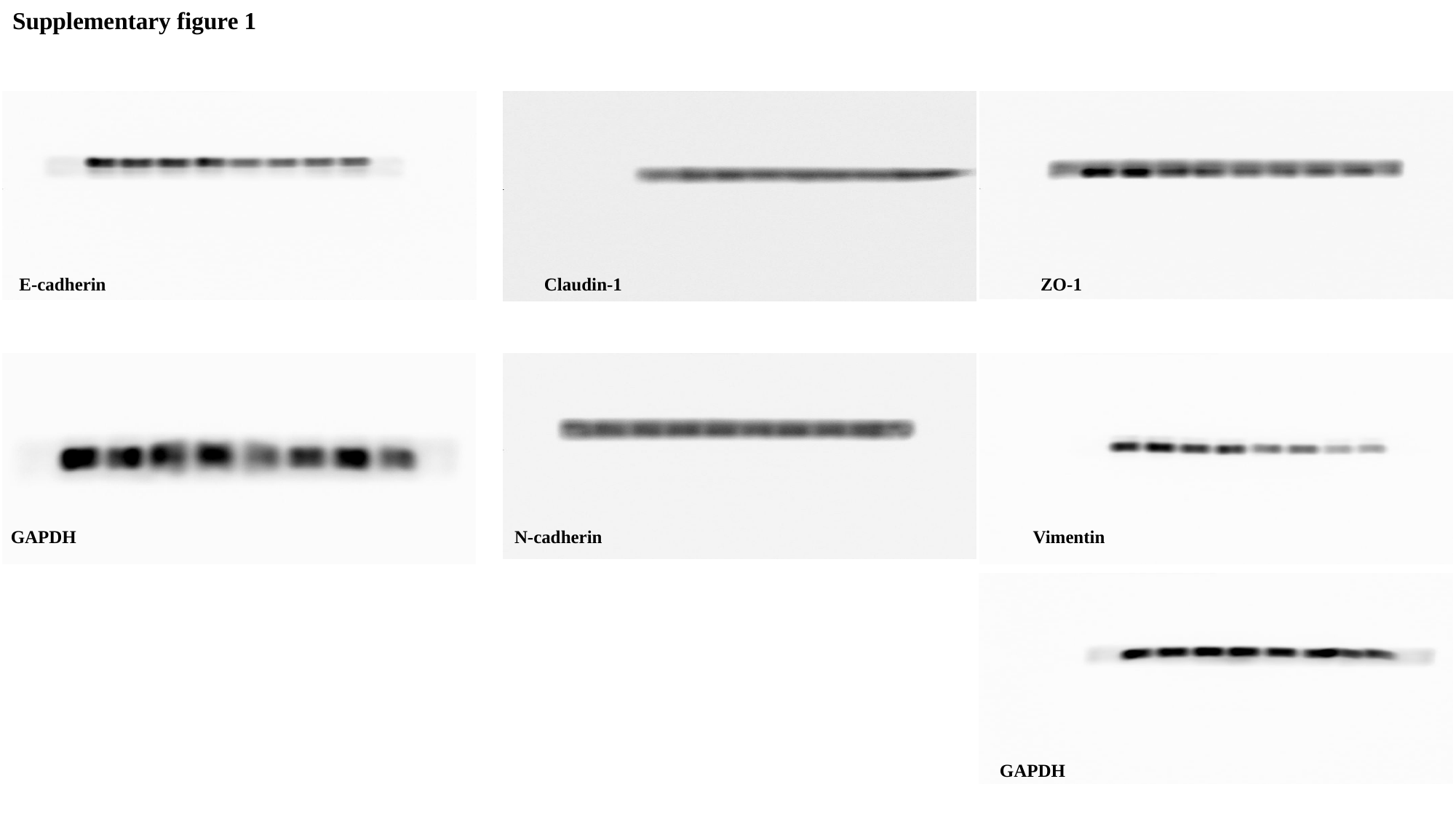

Supplementary figure 1
E-cadherin
Claudin-1
ZO-1
GAPDH
N-cadherin
Vimentin
GAPDH

## Slide 6
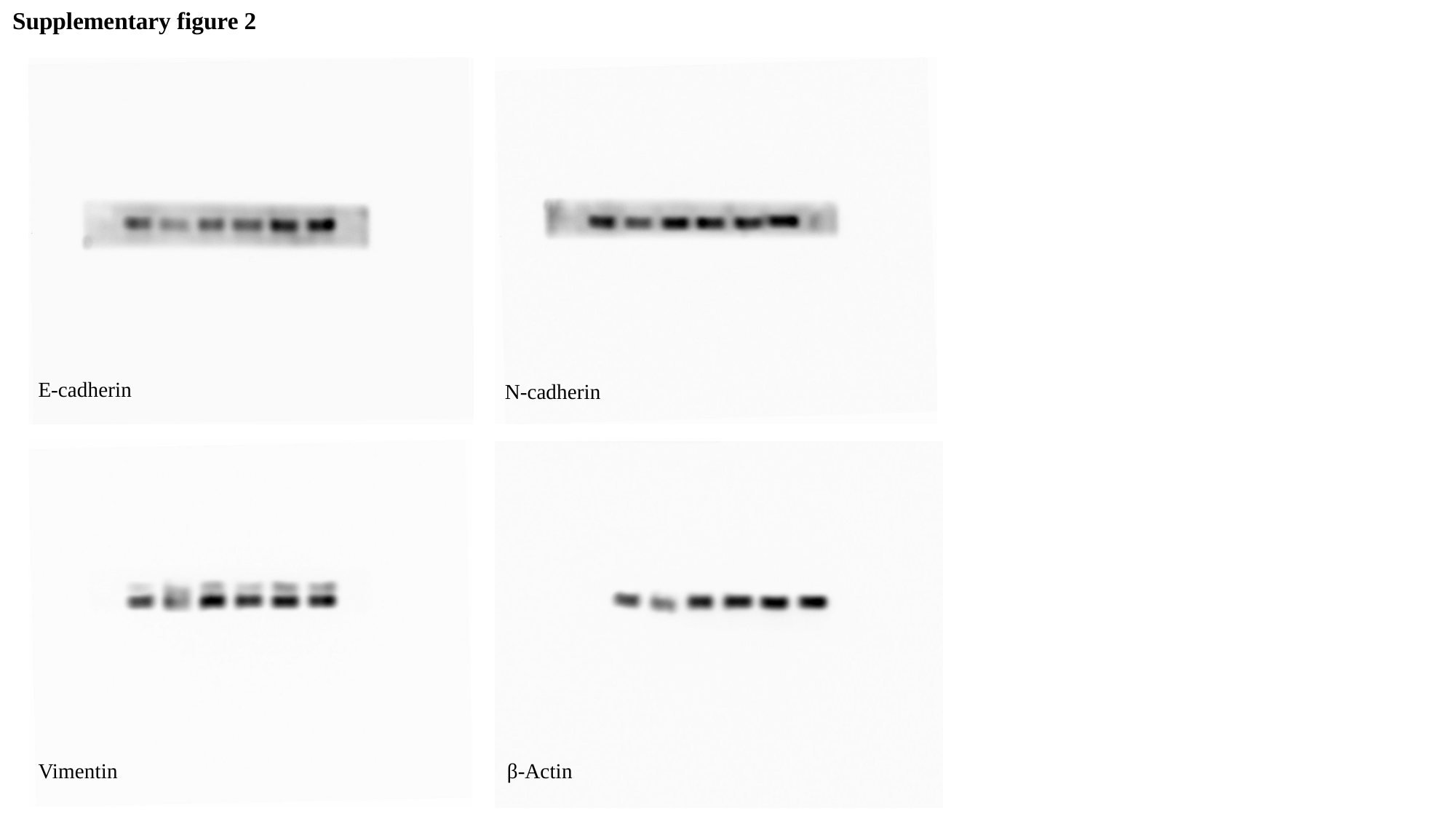

Supplementary figure 2
E-cadherin
N-cadherin
Vimentin
β-Actin
